# Relationship between Chemical Composition and Nematicidal Activity of Different Essential Oils

**DOI:** 10.3390/plants9111546

**Published:** 2020-11-11

**Authors:** Trifone D’Addabbo, Maria Pia Argentieri, Sebastiano Laquale, Vincenzo Candido, Pinarosa Avato

**Affiliations:** 1 Institute for Sustainable Plant Protection, National Council of Research, 70126 Bari, Italy; sebastianolaquale@yahoo.it; 2 Department of Pharmacy-Drug Sciences, University of Bari Aldo Moro, 70125 Bari, Italy; mariapia.argentieri@uniba.it (M.P.A.); pinarosa.avato@uniba.it (P.A.); 3 School of Agricultural, Forest, Food and Environmental Sciences, University of Basilicata, 85100 Potenza, Italy; vincenzo.candido@unibas.it

**Keywords:** essential oils, bioactive components, nematicidal activity, *Meloidogyneincognita*, sustainable management

## Abstract

In this study, the relationship between nematicidal activity and chemical composition of ten essential oils (EOs) from different plant species was investigated both in in vitro assays on juveniles (*J2*) and eggs of the root-knot nematode *Meloidogyne incognita* and in experiments on tomato in soil infested by *M. incognita*. Nematode *J2* were exposed for 4, 8 or 24 h to 0.78–100 μg mL^−1^ concentrations of each EO, whereas 24, 48 or 96 h exposures to 250, 500 and 1000 μg mL^−1^ solutions were tested on *M. incognita* egg masses. Treatments with 50, 100 or 200 μg kg soil rates of each EO were applied in the experiment on potted tomato. The highest nematicidal potential resulted for the *C. verum* EO, as highly toxic to both *M. incognita* *J2* and eggs and strongly suppressive on nematode multiplication on tomato roots. The infestation of *M. incognita* on tomato roots was also strongly reduced by the EOs from *E. citriodora* and *S. aromaticum*, both highly toxic to *M. incognita* *J2* but less active on nematode eggs. Adversely, *R. graveolens* EO strongly inhibited the egg hatch but was limitedly toxic to the infective *J2*. Chemical composition of the EOs was determined by GC-FID and GC-MS. The ten EOs showed a very different chemical composition in terms of major phytochemicals, with one or two dominant components totally amounting up to 85%. The structure–activity relationship based on the main phytochemicals identified in the assayed EOs and their nematicidal effects on *M. incognita* was also discussed. Results from this study confirmed that the selection of suitable EO raw materials can lead to the formulation on new effective nematicidal products.

## 1. Introduction

The management of root-knot nematodes (*Meloidogyne* species) represents a serious issue in many agricultural systems due to the heavy yield losses caused to a large number of herbaceous and fruit crops [1]. Criteria of environmental sustainability imposed by EU politics and public opinion have been reducing the use of synthetic nematicides in favour of more sustainable nematode management strategies [2], such as the use of plant-derived nematicidal compounds [3]. A large number of plant products have been tested for their nematicidal properties throughout the past decades, though only few, such as derivatives of Brassicaceae plants or neem (*Azadirachta indica* Juss) have been converted into effective industrial nematicidal formulates [4,5,6].

In recent years, researchers’ attention focused on a further promising source of botanical nematicides, such as the essential oils (EOs) produced by a large number of aromatic and medicinal plants, as extensively proved for a biocidal activity on phytoparasitic nematodes also including root-knot species [7,8].

EOs are composite mixtures of different classes of chemical compounds, mainly monoterpenes and sesquiterpenes, with a variable activity on phytoparasitic nematodes and targeting different nematode metabolic sites [8,9,10]. Investigation of structure–activity relationships regulating EOs’ effects against these phytoparasites is a key issue for their application as nematicidal products, as their nematotoxic effectiveness strictly depends on their quanti-qualitative composition and synergistic or antagonistic interactions among present components [11]. Laquale et al. [7] related the strong suppressiveness to the root-knot nematode *Meloidogyne incognita* Kofoid et White (Chitw.) of two EOs from *Monarda didyma* L. and *M. fistulosa* L. to their content of carvacrol and thymol, as repeatedly confirmed for a high nematicidal activity on *Meloidogyne* species [10,12,13,14,15]. Adversely, the low activity on *M. incognita* of an EO from *Citrus sinensis* (L.) Osbeck was attributed to the poor activity of the EO dominant component, limonene [15].

This study focused on the EOs from ten plant species of different botanical and geographical origin, i.e., *Cinnamomum camphora* (L.) J. Presl. (camphorwood), *C. verum* J. Presl. (cinnamom), *Eucalyptus citriodora* Hook (lemon eucalyptus), *E. globulus* Labill. (blue gum), *Mentha piperita* L. (peppermint), *Pelargonium asperum* Ehrh. ex Willd. (bourbon geranium), *Ruta graveolens* L. (common rue), *Schinus molle* L. (false pepper) and *Syzygium aromaticum* (L.) Merr. et Perry (clove). Most of these EOs were already documented for a nematicidal activity, though generally based on limited in vitro bioassays and without any specific correlation to their compositional profile. An in vitro activity on infective root-knot nematode juveniles (*J2*) and/or eggs was already documented for the EOs of *C. verum* [16,17], *E. citriodora* [12,18], *M. piperita* [10,18], *P. asperum* [10,18,19], *R. graveolens* [20,21] and *S. aromaticum* [22]. Moreover, soil treatments with some of these ten EOs were also documented for variable effects on *M. incognita* infestation on tomato [23,24].

The aim of this study was a comparative investigation of the nematicidal potential of these ten EOs against *M. incognita* through both in vitro bioassays on *M. incognita* infective *J2* and eggs and an experiment in soil on potted tomato as well as to elucidate the relationship between nematicidal activity and chemical structure of each EO.

## 2. Results

### 2.1. Chemical Composition of the EOs

**Figure 1:**
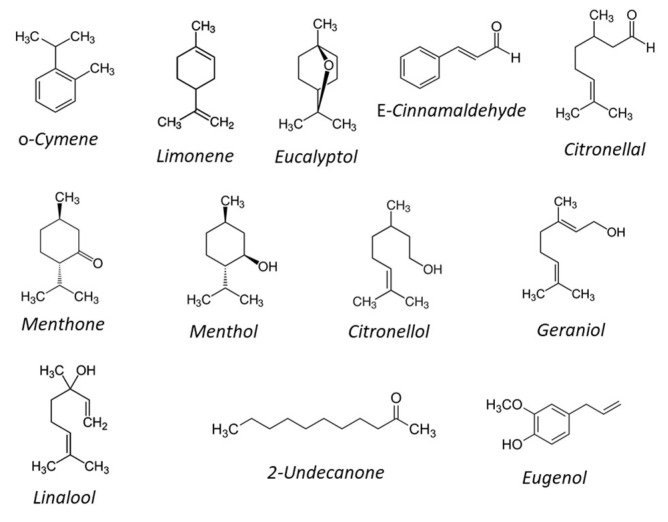
Chemical structure of main constituents of the ten EOs.

Each of the ten EOs presented a distinctive chemical profile of major components (Table 1; Figure 1).

Thus, both EOs from the two Cinnamomum species, *C. camphora* and *C. verum*, contained only few constituents but showed an extremely different compositional pattern. The *C. camphora* EO was characterized by terpenes, with limonene (59.3%), eucalyptol (21.8%) and o-cymene (16.8%) as the most abundant components, while the EO from *C. verum* contained phenolics such as E-cinnamaldehyde (84.8%) and eugenol (13.4%). A different chemical composition was also shown by the EOs from the two Eucalyptus species, *E. citriodora* and *E. globulus*. Citronellal (83.9%) was the main constituent of *E. citriodora* EO, whereas eucalyptol (91.7%) was almost the only component of the EO from *E. globulus*. In both these two EOs, very small amounts of other monoterpenoids were also detected. Limonene was the dominant component of *C. aurantium* EO, accounting for 94.9% of the total EO, though small amounts of β-myrcene (1.6%), linalool (1.0%) and linalyl acetate (1.5%) were also detected. The sample of *M. piperita* EO contained mainly menthane-type monoterpenoids, with menthol (54.8%), menthone (20.5%), iso-menthone (11.3%) and menthyl acetate (4.0%) making up the bulk of constituents. The EO from *P. asperum* was a mixture of several monoterpenoids, with citronellol (35.0%), geraniol (22.1%) and linalool (12.8%) as major components. The dominant component of the R. graveolens EO was the aliphatic ketone 2-undecanone (83.2%), with a very poor content of terpenoids but carvacrol (15.0%). The EO of *S. molle* was a mixture of several mono- and sesquiterpenes, with α-pinene (14.7%), eugenol (12.0%) and linalool (10.0%) as main monoterpene constituents and cedryl acetate (8.8%) and *β*-caryophyllene (5.4%) as the two main sesquiterpenes. Finally, only eugenol (89.6%) and the two sesquiterpenes β-caryophyllene (8.0%) and α-humulene (2.4%) were detected in the EO from *S. aromaticum*.

### 2.2. Juvenile Mortality Assay

**Figure 2:**
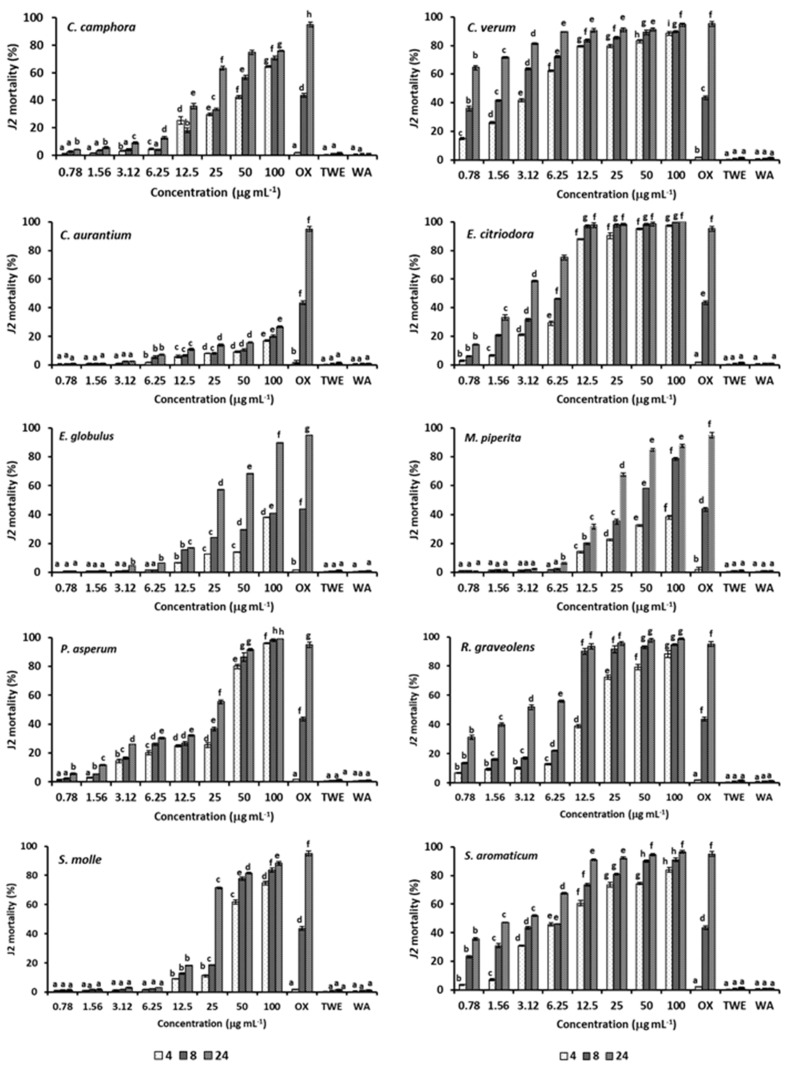
Mortality (%) of *M. incognita J2* after 4, 8 or 24 h exposure to a 0.78–100 μg mL^−1^ range of concentrations of the ten tested EOs. Data are means of four replicates. At each exposure time, bars marked with the same letter are not significantly different (*p* ≤ 0.05) according to the least significant difference test.

The EOs from the two Cinnamomum species showed a different activity on *M. incognita* J2, as a 64% mortality rate was reached after 24 exposure to 0.78 and 25 μg mL^−1^ solutions of *C. verum* and *C. camphora* EOs, respectively (Figure 2).

In addition, both 4 and 8 h treatments with the C. verum EO were also significantly more active than the nematicide Oxamyl at almost all the tested concentrations. Toxicity to *M. incognita J2* largely differed also between the two Eucalyptus EOs, as the *E. citriodora* EO caused an almost complete mortality even after 8 h exposure to a 12.5 μg mL^−1^ concentration while the EO from *E. globulus* peaked a 90% J2 mortality only at the maximum concentration x time combination. The EO from *P. asperum* was strongly toxic at the 50 and 100 μg mL^−1^ solutions but limitedly active at lower concentrations. At a less instance, a similar performance was provided also by the EOs of *M. piperita* and *S. molle*, as both causing more than 80% mortality only at the two highest concentrations. Mortality of *J2* ranged 31–56% after a 24 h immersion in 0.78–6.25 μg mL^−1^ solutions of the *R. graveolens* EO, but raised above 90% since 8 h exposure to a 12 μg mL^−1^ concentration. The EO of *S. aromaticum* resulted in highly toxic to *M. incognita J2*, as 24 h exposure caused 28.7–31.0% mortality rates even at 0.78 and 1.56 μg mL^−1^ EO concentrations, respectively, as well as an almost complete mortality (95–97%) at the two highest concentrations. Adversely, the *C. aurantium* EO was poorly active on nematode *J2*, as causing only 26.7% mortality after 24 h exposure to the 100 μg mL^−1^ solution. At all the exposure times, the lowest LC_50_ values were calculated for the *C. verum* EO, as confirming the highest activity of this EO within the ten EO samples in comparison (Table 2). Low LC_50_ values were also estimated for the 24 h treatment with the EOs from *S. aromaticum*, *E. citriodora* and *R. graveolens* EOs, 1.8, 1.6 and 1.4 μg mL^−1^, respectively. LC_50_ values of the C. aurantium EO always resulted largely above the range of tested concentrations, as confirming the poor activity on nematode *J2*.

### 2.3. Egg Hatchability Bioassay

Hatchability of *M. incognita* eggs was strongly affected by the EOs from *R. graveolens* and *C. verum*, as reduced to only 1.2 and 7.0%, respectively, after 96 h egg mass exposure to a 500 μg mL^−1^ solution, and also resulted always significantly lower than Oxamyl (Table 3). Compared to *C. verum*, *R. graveolens* EO solutions were largely more toxic to nematode eggs at the lowest concentration, but significantly less active at 500 and 1000 μg mL^−1^. The EOs from *E. citriodora*, *P. asperum* and *S. molle* reduced egg hatchability to 54–56% and 40–43% only after a 96 h egg mass exposure to the 500 and 1000 μg mL^−1^ solutions, respectively. Analogously, the *E. globulus* EO resulted in a discrete reduction of nematode egg hatchability only at the maximum concentration x time combination. Limited activity on *M. incognita* eggs was recorded also for the *S. aromaticum* EO, as 70.5 and 54.2% cumulative hatch values were found even after an egg mass exposure to a 1000 μg mL^−1^ solution for 24 and 48 h, respectively. Finally, egg hatchability was poorly affected by *M. piperita* and *C. aurantium* EOs, as not significantly different or only slightly lower compared to the non-treated control.

### 2.4. Experiment in Soil

All the soil treatments with the ten EOs significantly suppressed *M. incognita* multiplication and gall formation on tomato roots in comparison with the non-treated control, though always resulting significantly less suppressive than the nematicide Oxamyl (Table 4). Soil treatments with the EOs of *S. aromaticum*, *C. verum* and *E. citriodora* resulted in the lowest number of nematode eggs and J2 on tomato roots from, whereas *P. asperum* and *R. graveolens* EOs were mainly active on gall formation. The EOs from *C. aurantium* and *M. piperita* confirmed their poor activity also in soil, as resulting in the lowest suppressive effect on the root-knot nematode infestation.

Most treatments with the ten EOs also caused a significant increase of tomato aerial and root biomass (Table 4). Growth effect of the EOs of *C. verum* and *E. citriodora* was significantly larger than that of the other EOs and, at high application rates, also of Oxamyl. Adversely, the lowest tomato growth performance was always recorded in soil treated with the *C. aurantium* and *M. piperita* EOs.

### 2.5. Analysis of Aggregated Data

**Figure 3:**
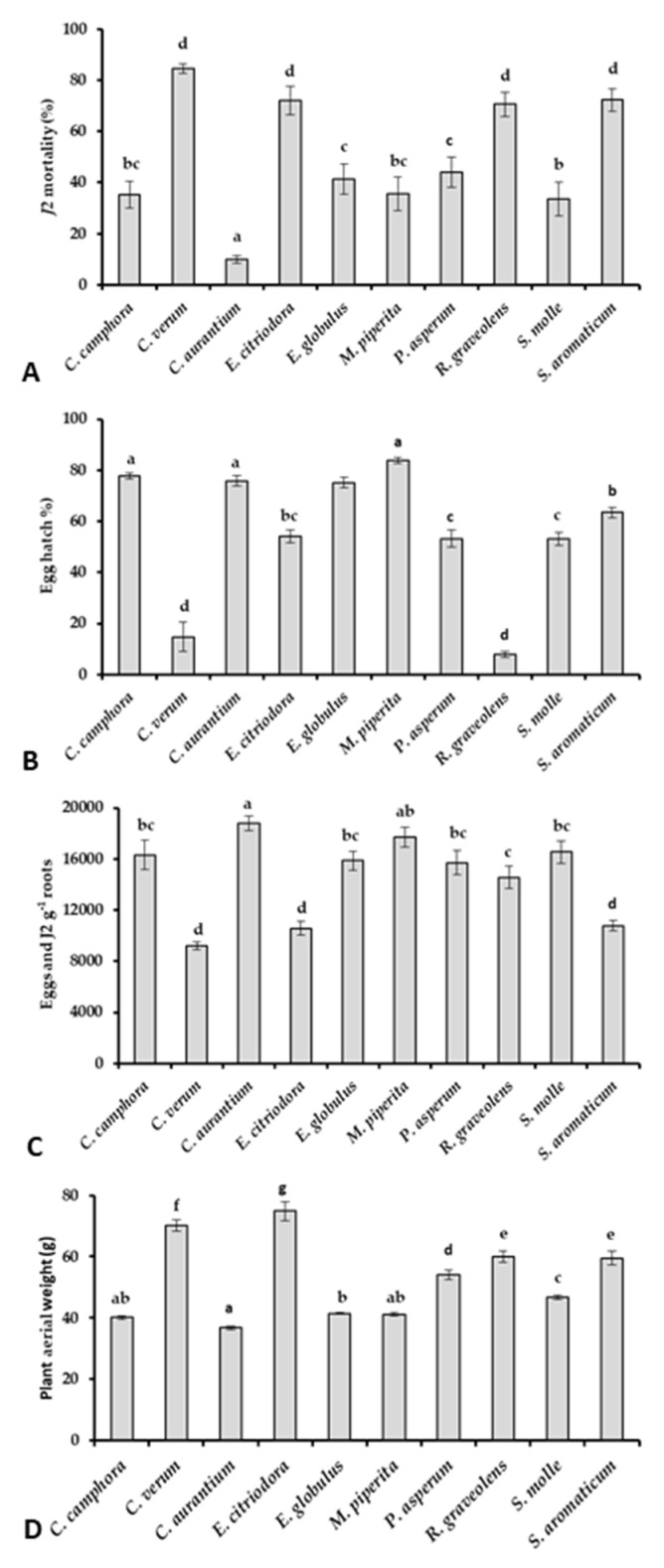
EOs’ aggregated effects on *J2* mortality (**A**), egg hatchability (**B**), *M. incognita* multiplication on roots (**C**) and tomato growth (**D**). Bars marked with the same letters are not significantly different according to Least Significant Difference Test (*p* ≤ 0.05).

A summary analysis of aggregated data showed that the highest larvicidal activity was provided by the EOs from *C. verum*, *E. citriodora*, *R. graveolens* and *S. aromaticum*, whereas the ovicidal effect of *C. verum* and *R. graveolens* EOs was largely higher than that of the other EOs (Figure 3).

In the experiment in soil, the highest aggregated suppressiveness to *M. incognita* was shown by the EOs of *C. verum*, *E. citriodora* and *S. aromaticum* followed by the *R. graveolens* EO. In addition, the EOs from *E. citrodora* and *C. verum* and, at a lesser instance, those from *R. graveolens* and *S. aromaticum* showed also the strongest effect on plant growth.

The lowest in vitro and in vivo nematicidal performance, as well as the lowest plant growth effect, were summarily found for the EOs of *C. aurantium* and *M. piperita*.

## 3. Discussion

Data displayed in this study evidenced strong differences among the nematicidal activity of the tested EOs and also when derived from plant species of the same genus. Within the two *Cinnamomum* EOs, the *C. verum* EO was largely more active than that from *C. camphora* both on *in vitro J2* and eggs as well as more suppressive on *M. incognita* infestation on tomato. These findings are in accordance with the literature data, as in previous in vitro comparative studies on a wide range of different EOs, *C. verum* EO always showed the most potent nematicidal activity both on the pinewood nematode *Bursaphelenchus xylophilus* (Steiner et Buhrer) Nickle [25,26] and root-knot nematodes *M*. *incognita* [16] and *M. graminicola* Golden and Birchfield [17]. In addition, soil treatments with *C. verum* EO resulted in a strong suppression of *M. incognita* infestation on tomato also in a previous preliminary study of our group [23], and were found for a significant reduction of root gall formation on rice seedlings in soil infested by *M. graminicola* [17]. Interestingly, aqueous extracts of *C. verum* were also documented for causing a complete *J2* mortality and a strong egg hatch inhibition on the root-knot nematode *M. exigua* Goeldi [22]. Adversely to that of *C. verum* EO, the nematicidal activity of the EO from *C. camphora* is poorly documented, as limited to the report of a moderate suppressiveness on *M. incognita* on tomato [23]. However, soil treatments with *C. camphora* ethanolic extracts in greenhouse were found to significantly suppress the infestation of *M. javanica* (Treub) Chitwood on greenhouse tomato, and to reduce the incidence and severity of wilt disease complex due to root-knot nematode association with the fungus *Fusarium oxysporum* f.sp *lycopersici* Snyder et Hansen [27].

The EO from *C. verum*, as prevalently constituted by *E*-cinnamaldehyde, had a composition consistent with that previously described [28], while the composition of the EO from *C. camphora* resulted quite unusal for the high content of limonene along with eucalyptol, which instead characterizes some chemotypes of this species [29]. Plant EOs have been reported to have a large number of biological activities which are determined by their specific chemical composition. Thus, the highest nematicidal efficacy of *C. verum* EO can be likely attributed to the presence of *E*-cinnamaldehyde as its major component. The toxicity of this compound and/or EOs containing *E*-cinnamaldehyde to different pests, including nematodes, has been demonstrated in several studies and related to its aldehydic structure (Figure 1). In specific investigations [26,30], *E*-cinnamaldehyde resulted in fact more active as nematicide or acaricide than other cinnamic acids characterized by different functional groups. Analogously, the lower nematicidal performance of the *C. camphora* EO was expected on the base of its chemical composition. We have in fact previously shown [15] that limonene, the main phytochemical of the EO from *C. camphora*, has a very poor nematicidal activity, independently of the nematode species. In addition, a limited nematicidal activity on *M. incognita* was also shown for an EO from *C. sinensis* also containing limonene as dominant component [15]. Limonene is an aliphatic hydrocarbon and the lack of any functional group in its structure might possibly reduce its reactivity.

The strong nematicidal activity of the *E. citriodora* EO is also in agreement with literature data. An *E. citriodora* EO was reported for the highest toxicity to *M. incognita J2* in an in vitro bioassay on eight different EOs [18], and EOs and extracts from different *Eucalyptus* species, also including *E. citriodora*, were found to reduce root-knot nematode *J2* mobility and viability and egg hatchability [12]. Moreover, soil fumigation with an *E. citriodora* EO resulted in a strong reduction of *M. incognita* multiplication and egg formation on tomato roots [24]. In the same study, the fumigant activity of *E. globulus* EO on *M. incognita* was consistently lower than that of an EO from *E. citriodora*, as confirming the different nematicidal effectiveness of the two *Eucalyptus* EOs observed in this study.

In agreement with literature findings [24], the bulk of the tested *E. citriodora* EO was made up by aliphatic terpenes such as citronellal, the major compound (83.9%), citronellol and citronellyl formate, while *E. globulus* was prevalently (92%) constituted by the byciclic ether eucalyptol. As for the two terpenes described above, the higher nematicidal activity of *E. citriodora* EO can be ascribed to the functional groups present in citronellal (aldehydic function) and its derivatives compared to eucalyptol (Figure 1). In specific studies aimed to evaluate the toxicity of a series of terpenes against bacterial and microbial systems [31,32], it was found that the presence of an oxygen-related function (as in citronellal and citronellol) and a double bond (as in citronellal) contributes to enhance the reactivity of the molecule towards biological processes involving the transfer of electrons by increasing its electronegativity.

In the present study the EO of *P. asperum* was found more active on *M. incognita J2* than in previous in vitro bioassays, which reported for this EO a significant toxicity to *M. incognita J2* only at high concentrations and/or exposure times [18,19]. Moreover, a weak in vitro sensitivity to *P. asperum* EO was also described for the infective *J2* of *M. javanica* [10]. Adversely, the satisfactory suppressiveness to *M. incognita* on tomato of soil treatments with the *P. asperum* EO seems to be in good agreement with the effects previously shown in literature [23].

The *P*. *asperum* EO used in this study was a complex mixture of terpenes prevalently made up by three major components, linalool (12.8%), citronellol (35.0%) and geraniol (22.1%), as agreeing with previous literature data [24,33]. These compounds are all characterized by a hydroxyl function considered to be crucial for reactivity towards biological systems (Figure 1). However, in spite of the structural features of its components, the *P. asperum* EO showed a lower nematicidal activity compared to *C. verum*, *E. citriodora*, *R. graveolens* or *S*. *aromaticum* EOs, all characterized by only one major component accounting for 83–89%. This result may suggest that constituents of *P. asperum* EO do not act synergistically.

The EO of *R. graveolens* was particularly toxic to *M. incognita* eggs, as almost completely inhibiting egg hatch, though strongly active on nematode *J2* only at 24 h exposure. Previous in vitro studies also indicated a strong activity of *R. graveolens* EO on the root-knot nematode *M. chitwoodi* Golden, O’Bannon, Santo and Finley [21] and *B. xylophilus* [34], whereas da Silva et al. [20] reported high mortality rates of *M*. *incognita J2* only at high (500–1000 μg mL^−1^) *R. graveolens* EO concentrations. In the present study, the *R. graveolens* EO was less suppressive to *M. incognita* on tomato than the EO of *E. citriodora*, in agreement with the effects of soil biofumigation with the same two EOs reported by Laquale et al. [24].

As previously documented [20,24], the EO of *R. graveolens* is very poor in terpenoids except carvacrol, while the dominant component is a dialkyl ketone, 2-undecanone. This compound can structurally form a keto-enol tautomer and then can be considered as an oxygenated highly reactive hydrocarbon responsible for toxicity to nematodes (Figure 1). In addition, the EO of *R. graveolens* is also characterized by a discrete amount of carvacrol, a monoterpenoid phenol with many biological properties [10,12,35,36,37], that can likely act in a synergistic way with *2*-undecanone. Moreover, this phytochemical is an isomer of thymol, which was also shown highly toxic to *M. incognita* [15].

The high toxicity to *J2* and, at a lesser instance, eggs of *M. incognita* of the *S. aromaticum* EO also agrees with literature studies, as EOs and extracts from this plant species were previously documented for a high *J2* mortality and a strong egg hatch inhibition on *M. exigua* [22], as well as for a nematicidal activity on different stages of *B. xylophilus* [25]. In our experiment in soil, the *S. aromaticum* EO resulted one of the most effective at reducing *M. incognita* infestation on tomato roots, in accordance with the high suppressive performance previously reported for soil drench treatments with *S. aromaticum* EO water emulsions [23]. In contrast, Meyer et al. [38] did not find any consistent reduction of *M. incognita* infestation on different greenhouse vegetable crops by not phytotoxic concentrations of a *S. aromaticum* EO formulation resulted highly active in preliminary lab assays.

As described by previous studies [39], the EO from *S. aromaticum* was almost completely made up by eugenol (89.6%), which should be then considered as the main responsible of its nematotoxic activity. Eugenol is a 2-alkyl(oxy)phenol which shares some chemical features with the above discussed phenolic monoterpenes carvacrol and thymol, also reported as very active against bacteria [32] and insects [40]. As demonstrated in a specific study aimed to evaluate structure–activity relationships of eugenol and its congeners against some insects, eugenol allylic double bond and phenolic proton are essential for the molecule bioactivity [41]. A neurotoxic mode of action has been highlighted specifically for some compounds such as eugenol. Docking experiments have in fact shown that this phytochemical may act as an acetylcholinesterase inhibitor and can block octopamine receptors, thus supporting a nematicidal action based on nervous system targets [42,43]. However, mechanisms of action of EOs and their components against nematodes are still debated and further hypotheses, such as an effect on membranes permeability, can be found in literature studies [8,10].

Effects of the *S. molle* EO on *M. incognita J2* and eggs and on nematode infestation on tomato were significant only at high solution concentrations and soil application dosages, respectively. The EO from *S. molle* was previously documented for antifungal and antibacterial properties [44] as well as for insecticidal and acaricidal activities [45,46]. The only literature report of a *S. molle* EO nematicidal activity described a higher suppressiveness to *M. incognita* on tomato compared to that emerged from this study [23], whereas there are no previous data on an in vitro toxicity to nematode *J2* and eggs. The tested EO of *S. molle* was a mixture of several monoterpenes and sesquiterpenes without any dominant compound, as also documented by literature data [47]. Thus, its effect on nematodes may reasonably be ascribed to a synergistic activity of all its components. Indeed, the *S. molle* EO was among the less active in this study and, according to above considerations, this result could be attributed to the high presence of terpenes without reactive functional groups. The EO of *S. molle* also contains low amounts of carvacrol and eugenol, 3.7 and 12.0%, respectively, but their expected bioactivity did not seem to emerge and to be determinant against *M. incognita.*

The EOs from *M. piperita* and *C. aurantium* showed the lowest activity on *M. incognita* both in the in vitro assays and in the experiment in soil. The low activity of our *M. piperita* EO confirms the literature data, which generally documented a limited in vitro toxicity of this EO to root-knot nematode *J2* and eggs [10,18]. Similarly, a poor nematicidal effect of soil treatments with *M. piperita* EO solutions was reported also in a study of Walker and Melin [48], as describing not significant effects of *M. piperita* EO in soil infested by *M. arenaria* on tomato. Our sample of *M. piperita* EO contained menthane-type monoterpenoids, with menthol and menthone as the two dominant compounds (54.8 and 20.5%, respectively), as also documented in previous studies [24,49]. Therefore, the low nematicidal effect of *M. piperita* EO in our studies can be considered quite unexpected, as the structural features of the above two terpenes could adversely suggest a high nematicidal effectiveness.

This study is the first investigation of the nematicidal properties of *C. aurantium* EO, whereas it was widely proved for a biocidal and/or repellent activity on crop insect pest insects, such as *Bemisia tabaci*, or stored product parasites as *Callosobruchus maculatus* [50,51]. Our *C. aurantium* EO confirmed the typical chemical composition documented by previous literature reports [51], characterized by the presence of limonene as the almost exclusive component (94.9%). Therefore, according to the considerations above reported for the nematicidal properties of the *C. camphora* EO and limonene, the poor nematicidal activity of *C. aurantium* EO is largely expected.

## 4. Materials and Methods

### 4.1. Essential Oils

The analyzed samples consisted in ten commercially available (Erboris Orientis Dacor, Milan, Italy) distilled pure EOs from *Cinnamomum camphora* (L.) J. Presl. (camphorwood), *C. verum* J. Presl. (cinnamon), *Eucalyptus citriodora* Hook (lemon eucalyptus), *E. globulus* Labill. (blue gum), *Mentha piperita* L. (peppermint), *Pelargonium asperum* Ehrh. ex Willd. (bourbon geranium), *Ruta graveolens* L. (common rue), *Schinus molle* L.(false pepper) and *Syzygium aromaticum* (L.) Merr. & L.M. Perry (clove).

### 4.2. Chemical Analysis of the EOs

A Trace gas chromatograph (GC)-FID Ultra Thermo Finnigan was used for the compositional analysis of the ten EOs. A sample (1µL) of each EO solubilized in hexane was injected in the cold on-column mode in a Agilent DB-5 (J&W Scientific, Milan, Italy) fused silica capillary column (30 m × 0.25 mm; 0.25 µm film thickness). Analytical conditions were as follows: Detector temperature 300 °C; column temperature was programmed from 60 °C (5 min isothermal) to 280 °C (30 min isothermal) at 4 °C/min. Hydrogen was the carrier gas (35 kP; 2.0 mL/min); data were processed using the Chrom-Card 32-bit computing software.

Gas chromatography-Mass spectrometry (GC-MS) analyses were performed with a Hewlett Packard 6890–5973 (Milan, Italy) mass spectrometer interfaced with a HP Chemstation (Milan, Italy). Chromatographic conditions were set as follows: Column oven program from 60 °C (5 min isothermal) to 240 °C (15 min isothermal) at 3 °C/min; injector, 280 °C. Helium was the carrier gas (flow rate, 1 mL/min). A HP-5 MS capillary column (30 m × 0.25 mm; 0.25 µm film thickness) was used. MS operating parameters were: Ion source, 70 eV; ion source temperature, 200 °C; electron current, 34.6 µA; vacuum 10^−5^ torr. Mass spectra were acquired over a 40–800 amu range at 1 scan/s. The ion source was operating in the electron impact mode. Samples (1µl) were injected using the splitless sampling technique.

### 4.3. Identification and Quantitation of the EOs Components

Chemical composition of the analysed EOs was achieved by comparison of GC retention times of their constituents with authentic reference compounds in combinations with arithmetic indexes (AI) and by means of reference mass spectra from standard compounds and/or from library files [15,52,53].

AI index values were calculated using an n-alkane series (C6–C32) under the same GC conditions as that for the samples. The relative amount of individual components of the oil were expressed as percent peak area relative to total peak area from the GC-FID analysis of the whole extracts without the use of correction factors. A linear proportion between the areas was used, assuming an equal response factor for all detected compounds.

### 4.4. Juvenile Mortality Bioassays

The Italian population of *M. incognita* used for both in vitro and in vivo experiments was collected from infested tomato roots in a field at Castellaneta (Taranto province, Apulia region) and then multiplicated on tomato (*cv*. Roma) plants at a constant 25 ± 2 °C temperature in a glasshouse. Mature nematode egg masses picked from the tomato roots were directly used in the hatching assay or incubated in distilled water at 25 °C in a growth chamber. The *J2* emerged during the incubation period were recovered and maintained in a refrigerator (5 °C) until their use in the toxicity bioassay.

### 4.5. Nematode Mortality Bioassays

A 0.5 mL volume of a distilled water suspension of *M. incognita J2* (about 200 specimens mL^−1^ water) was poured in 1.5 mL Eppendorf tubes and then added with the same volume of 1.56, 3.12, 6.25, 12.5, 25, 50, 100 and 200 μg mL^−1^ solutions of the ten EOS in a 0.3% Tween 20 water solution, as to reach 0.78, 1.56, 3.12, 6.25, 12.5, 25, 50 and 100 μg mL^−1^ treatment EO concentrations. Nematode suspensions were treated with each test solution for 4, 8 or 24 h, with four replicates of each concentration x exposure time combination. Distilled water, the 0.3% Tween 20 solution and a 2 mL L^−1^ water solution of a commercial formulation (Vydate^®^ 10 L) of the nematicide Oxamyl (10% a.i.) were also included as controls. The *J2* from each tube were microscopically checked for their motility at the end of each exposure time and then transferred into distilled water for 72 h, after which persistence of immobility was stated as mortality. The Abbott’s formula *m* = 100 × (1 − *nt*/*nc*), in which *m* = percent mortality; *nt* = number of *J2* still viable after the treatment; *nc* = number of viable *J2* in water control was applied to calculate *J2* mortality rates [54].

### 4.6. Egg Hatchability Bioassays

Fifty *M. incognita* egg masses, ranging about 400 eggs per mass, were immersed in 0.5 mL of distilled water in 1.5 mL Eppendorf tubes and then added with the same volume of 500, 1000 and 2000 μg mL^−1^ 0.3% Tween 20 water solutions of each of the ten EOs, as to reach 250, 500 and 1000 μg mL^−1^ final concentrations. Distilled water and the 0.3% Tween 20 water solution were included as controls. All concentration x exposure time combinations and the two controls were replicated four times. After each treatment with EOs, the egg masses were repeatedly washed in distilled water and then transferred into 2 cm diameter sieves (215 μm aperture) placed in a 3.5 cm diameter Petri dish. The egg masses were then submerged by 3 mL of distilled water and placed into Petri dishes in a growth chamber at 25 °C. Number of emerged *J2* was microscopically counted and distilled water was renewed at weekly intervals throughout 5 weeks, after which the egg masses were shaked for 3 min in a 1% sodium hypochlorite solution [55] and released unhatched eggs were counted under a microscope. Egg hatchability was expressed as percentage of hatched eggs compared to the total (hatched + unhatched) egg content of egg masses.

### 4.7. Experiment in Soil

Roots from the infested tomato plants previously reared in a glasshouse were minutely cut and thoroughly mixed, then five 10 g samples were picked on which *M. incognita* egg and *J2* density was assessed by their extraction (55) followed by microscopical count. Steam sterilised soil (64.4% sand, 18.7% silt, 16.9% clay, 0.8% organic matter and 7.5 pH) was artificially infested with the root inoculum to reach up to a 20 eggs and *J2* mL^−1^ soil initial nematode population density and then poured into 1 L clay pots. Potted soil was then treated with 50, 100 and 200 µg kg^−1^ soil rates of each of the ten EOs carried by 400 mL volume of a 0.3% water solution of Tween 20. Infested soil, either non-treated or treated with a 2 mL kg^−1^ soil rate of the same commercial formulation of the nematicide Oxamyl used in the in vitro bioassay on nematode *J2* and sterilised soil were also included as controls. There were five replicates of each treatment, arranged in a randomised block design on the benches of a glasshouse maintained at 25 ± 2 °C constant temperature.

Three weeks after the treatments with EOs, the soil of each pot was transplanted with a 1-month-old tomato (cv. Roma) seedling. The tomato plants were uprooted two-months after the transplant, recording fresh weight of aerial parts and roots of each plant. Root gall formation caused by *M*. *incognita* infestation was evaluated on each tomato root according to a 0–5 scale (0 = no galls, 1 = 1–2 galls, 2 = 3–10 galls, 3 = 11–30 galls, 4 = 31–100 galls and 5 > 100 galls) [56]. Nematode multiplication was evaluated by extracting using the sodium hypochlorite method [55] and then microscopically counting eggs and *J2* from the roots of each replicate.

### 4.8. Data Analysis

The three experiments were repeated twice and, in the absence of significant experiment x treatment interactions, data from the two experimental runs were pooled [54]. Error variances were homogenized by arcsin-transforming the pooled data before their statistical analysis by the PlotIT 3.2 (Scientific Programming Enterprises, Haslett, MI, USA) software. One or two-way analysis of variance were performed and means were compared by Fisher’s Least Significant Difference Test at *p* ≤ 0.05. The LC_50_ values of each EO on *M. incognita J2* were calculated by probit analysis [54] of mortality data from the in vitro bioassay.

## 5. Conclusions

EOs are generally acknowledged for their strong nematicidal activity, but this study indicated that nematicidal potential can consistently vary among different EOs according to their chemical composition, and that not all EOs can be suitable to the formulation of new nematicidal products. Therefore, the choice of an EO candidate for this specific use should be always preceded by a chemical analysis of its constituents [57], also taking into account the quanti-qualitative variability of their composition related to agronomical and climatic factors as well as to the distilled plant parts.

A further indication is that the selection of EO raw materials for new potential nematicides should also consider the target nematode stage, as an EO could be strongly toxic to infective *J2* but less or poorly active on eggs.

Moreover, the final choice of nematicidal EO materials should also result from the evaluation of technical and economic aspects, such as the suitability for slow-release micro- or nanoencapsulated formulations easily distributable in field and a prompt availability at reasonable costs.

However, as clearly evidenced by Isman et al. [57], plant EOs have a large number of positive aspects that strongly recommend their industrial use for the production of new nematicides in spite of the complexity of their selection process. EOs and their components can be strongly active on phytonematodes but poorly toxic to mammals and, as highly volatile, environmental safe. In addition, the coexistence of multiple mechanisms of activity and metabolic target sites [30,36,42,43] can avoid the insurgence of resistant populations frequently occurring for synthetic nematicides. Finally, most of EOs can be abundantly purchased at low prices due to their large worldwide production and trade, as ensuring a regular supply to nematicide industries.

Finally, the recent registration of new nematicides based on mixtures of synthetically derived terpenes such as thymol and geraniol indicates that EOs can also represent a model for new synthetic formulations of highly active components as an alternative to direct use as industrial raw materials.

## Figures and Tables

**Table 1:** Chemical composition of the ten essential oils (EOs) as determined by GC-FID and GC-MS analyses.

Compounds	*AI tab ^a^*	*AI ^b^*	Amount% ± SD
*C. camphora*			
α-Pinene	932	932	1.0 ± 0.01
*o*-Cymene	1022	1023	16.8 ± 0.10
Limonene	1024	1025	59.3 ± 2.20
Eucalyptol	1026	1026	21.8 ± 2.10
Terpinen-4-olo	1174	1175	0.5 ± 0.01
α-Terpineol	1186	1186	0.36 ± 0.01
*C. verum*			
*Z*-Cinnamaldehyde	1217	1217	1.8 ± 0.06
*E*-Cinnamaldehyde	1267	1265	84.8 ± 0.29
Eugenol	1356	1356	13.4 ± 0.34
*C. aurantium*			
α-Pinene	932	932	0.6 ± 0.006
Sabinene	969	969	0.3 ± 0.001
β-Pinene	974	974	0.2 ± 0.002
β-Myrcene	988	988	1.6 ± 0.002
Limonene	1024	1025	94.9 ± 0.02
Linalool	1095	1095	1.0 ± 0.01
Linalyl acetate	1254	1255	1.5 ± 0.006
*E. citriodora*			
α-Pinene	932	932	1.4 ± 0.01
β-Pinene	974	974	1.0 ± 0.03
Eucalyptol	1026	1026	1.0 ± 0.20
Isopulegol	1145	1148	7.0 ± 0.15
Citronellal	1148	1150	83.9 ± 1.50
Citronellol	1223	1223	4.7 ± 1.71
Citronellyl formate	1271	1270	1.0 ± 0.02
β-Caryophyllene	1417	1417	1.0 ± 0.02
*E. globulus*			
α-Pinene	932	932	2.5 ± 0.02
β-Pinene	974	974	0.5 ± 0.02
β-Myrcene	988	988	0.6 ± 0.02
α-Terpinene	1014	1014	0.2 ± 0.02
*p*.Cymene	1020	1021	3.3 ± 0.02
Eucalyptol	1026	1026	91.7 ± 0.02
γ-Terpinene	1054	1055	0.7 ± 0.02
Terpinen-4-ol-acetate	1299	1298	0.2 ± 0.02
α-Terpinyl-acetate	1346	1345	0.3 ± 0.02
*M. piperita*			
α-Pinene	932	932	0.4 ± 0.01
β-Pinene	974	974	0.6 ± 0.01
Limonene	1024	1026	4.5 ± 0.01
Linalool	1095	1095	0.4 ± 0.01
iso-Pulegol	1145	1143	1.2 ± 0.03
Menthone	1148	1148	20.5 ± 0.08
iso-Menthone	1158	1156	11.3 ± 0.07
Menthol	1167	1167	54.8 ± 0.20
iso-Menthol	1179	1178	0.5 ± 0.01
Menthol neo-iso	1184	1182	0.2 ± 0.02
α-Terpineol	1186	1186	0.3 ± 0.04
Pulegone	1233	1232	0.7 ± 0.01
Carvone	1239	1239	0.6 ± 0.01
Menthyl-acetate	1294	1292	4.0 ± 0.03
*P. asperum*			
α-Pinene	932	932	1.0 ± 0.01
Eucaliptol	1026	1026	1.8 ± 0.05
Linalool	1095	1095	12.8 ± 0.27
*cis*-Rose oxide	1106	1108	1.3 ± 0.03
*trans*-Rose oxide	1122	1121	0.5 ± 0.02
Menthone	1148	1148	1.4 ± 0.02
iso-Menthone	1158	1156	5.5 ± 0.24
Citronellol	1223	1223	35.0 ± 0.37
Geraniol	1249	1249	22.1 ± 0.30
Cytronellyl formate	1271	1273	7.0 ± 0.16
Geranyl formate	1298	1296	3.8 ± 0.20
α-Copaene	1374	1373	0.5 ± 0.10
Geranyl acetate	1379	1379	0.5 ± 0.02
α-Bourbonene	1384	1386	1.0 ± 0.03
β-Caryophyllene	1417	1417	2.0 ± 0.01
trans-Bergamotene	1432	1433	1.2 ± 0.04
trans-Murola-3,5-diene	1451	1450	1.0 ± 0.04
Citronellyl tiglate	1656	1655	1.6 ± 0.05
*R. graveolens*			
Eucalyptol	1026	1026	0.4 ± 0.01
*2*-Nonanone	1087	1088	0.5 ± 0.02
Camphor	1141	1141	0.4 ± 0.01
*2*-Decanone	1190	1190	0.5 ± 0.04
*2*-Undecanone	1293	1294	83.2 ± 0.25
Carvacrol	1298	1299	15.0 ± 0.31
*S. molle*			
α-Pinene	932	932	14.8 ± 0.16
Sabinene	969	969	3.4 ± 0.05
β-Pinene	974	974	7.0 ± 0.10
β-Myrcene	988	988	1.0 ± 0.05
δ-3-Carene	1008	1008	5.5 ± 0.07
*p*.Cymene	1021	1021	6.7 ± 0.09
Limonene	1024	1024	7.6 ± 0.09
Eucalyptol	1026	1026	0.3 ± 0.04
Linalool	1095	1095	10.0 ± 0.17
Terpinene-4-ol	1174	1174	1.2 ± 0.03
α-Terpineol	1186	1186	1.0 ± 0.05
Linalyl acetate	1254	1255	7.3 ± 0.16
Carvacrol	1299	1299	3.7 ± 0.14
δ-Elemene	1335	1333	0.5 ± 0.01
Eugenol	1356	1356	12.0 ± 0.13
β-Caryophyllene	1417	1417	5.4 ± 0.07
γ-Curcumene	1481	1480	0.8 ± 0.003
Myristicin	1517	1518	1.7 ± 0.06
Eugenil acetate	1521	1522	1.3 ± 0.03
Cedryl acetate	1767	1766	8.8 ± 0.39
*S. aromaticum*			
Eugenol	1356	1356	89.6 ± 0.40
β-Caryophyllene	1417	1417	8.0 ± 0.40
α-Humulene	1452	1453	2.4 ± 0.01

^a^ Arithmetic indexes (AI)from library files (see 4.3)]; ^b^ AI calculated by GC relative to a homologous series of *n*-hydrocarbons (C_6_-C_32_) eluted on a DB-5 column.

**Table 2:** LD_50_ values of the ten tested EOs at 4, 8 and 24 h exposures of *M. incognita J2*.

EOs	4 h			8 h			24 h		
	LD_50_	95% Fiducial CI		LD_50_	95% Fiducial CI		LD_50_	95% Fiducial CI	
		Lower	Upper		Lower	Upper		Lower	Upper
*C. camphora*	63.3	34.1	117.6	53.9	28.7	101.1	22.9	13.1	40.0
*C. verum*	4.9	2.6	9.5	1.6	0.7	3.9	0.1	0.02	0.4
*C. aurantium*	1704.0	416.7	6967.4	1996.9	459.4	8681.7	447.3	154.1	1298.4
*E. citriodora*	7.6	5.0	11.6	3.9	2.7	5.8	2.4	1.6	3.6
*E. globulus*	264.8	112.5	623.7	470.6	141.5	1564.7	26.7	16.8	42.6
*M. piperita*	167.7	76.2	369.4	44.4	26.2	75.2	20.7	13.4	32.0
*P. asperum*	20.6	12.8	33.1	31.7	17.2	58.3	13.0	7.6	22.1
*R. graveolens*	13.9	8.3	23.4	6.4	3.9	10.3	2.3	1.4	4.0
*S. molle*	70.3	39.9	123.9	43.8	26.3	72.8	22.6	14.5	35.3
*S. aromaticum*	11.8	6.9	20.3	4.4	2.9	8.4	1.8	0.9	3.6

**Table 3:** Percentage hatchability (means of four replicates ± SE) of *M. incognita* eggs after 24, 48 or 96 h exposure of egg masses to 125–1000 μg mL^−1^ solutions of the ten tested EOs.

Concentrations (μg mL^−1^)	Exposure Time (h)		
	24	48	96
*C. camphora*			
250	93.0 ± 1.7	83.7 ± 0.7	81.7 ± 0.6
500	87.7 ± 0.8	82.7 ± 0.9	77.5 ± 1.2
1000	85.2 ± 1.4	81.0 ± 0.9	73.5 ± 1.6
*C. verum*			
250	69.5 ± 2.1	52.5 ± 1.6	42.0 ± 2.2
500	11.0 ± 0.8	2.7 ± 0.5	1.2 ± 0.2
1000	1.7 ± 0.5	1.2 ± 0.2	1.0 ± 0.4
*C. aurantium*			
250	88.5 ± 0.8	85.0 ± 1.3	80.2 ± 1.2
500	85.0 ± 1.6	84.0 ± 0.4	79.5 ± 1.9
1000	83.5 ± 1.2	79.2 ± 2.6	67.5 ± 2.2
*E. citriodora*			
250	83.7 ± 1.8	68.5 ± 1.7	63.2 ± 1.6
500	75.7 ± 0.6	64.0 ± 1.3	55.7 ± 2.2
1000	67.7 ± 1.1	60.5 ± 1.0	43.2 ± 1.2
*E. globulus*			
250	86.5 ± 0.6	83.5 ± 1.0	81.5 ± 2.2
500	84.7 ± 0.7	81.2 ± 1.4	77.2 ± 1.4
1000	84.2 ± 1.4	76.5 ± 1.2	66.7 ± 1.5
*M. piperita*			
250	93.5 ± 0.6	90.2 ± 0.9	88.5 ± 0.3
500	92.5 ± 0.9	89.0 ± 1.5	84.0 ± 1.2
1000	90.0 ± 1.9	85.2 ± 0.6	79.0 ± 1.1
*P. asperum*			
250	82.2 ± 1.3	76.2 ± 0.7	65.5 ± 2.7
500	71.2 ± 1.1	68.5 ± 0.9	54.5 ± 0.9
1000	56.2 ± 5.9	52.0 ± 2.2	39.7 ± 1.1
*R. graveolens*			
250	26.7 ± 1.7	20.0 ± 0.4	13.5 ± 0.3
500	22.7 ± 0.6	16.0 ± 0.7	7.0 ± 0.4
1000	18.5 ± 1.4	5.0 ± 0.7	3.2 ± 0.6
*S. molle*			
250	79.5 ± 1.3	74.0 ± 0.4	62.2 ± 0.8
500	70..7 ± 1.3	65.5 ± 1.0	53.7 ± 1.6
1000	65.7 ± 1.4	59.7 ± 0.8	43.2 ± 1.3
*S. aromaticum*			
250	82.5 ± 0.9	76.0 ± 1.2	68.0 ± 0.9
500	77.5 ± 0.9	70.0 ± 1.8	68.0 ± 1.6
1000	70.5 ± 1.2	62.5 ± 1.4	54.2 ± 1.1
Oxamyl	86.2 ± 0.8	77.5 ± 0.6	66.2 ± 1.5
Tween 20	94.2 ± 0.6	92.7 ± 0.8	92.2 ± 0.8
Non treated	93.2 ± 1.3	93.2 ± 1.3	93.2 ± 1.3
LSD 0.05	4.5	3.3	3.9

**Table 4:** Effects (means of five replicates ± SE) of soil treatments with different rates of the ten tested EOs on the infestation of the root-knot nematode *M. incognita* and the growth of tomato (cv. Roma).

Dose (μg kg^−1^ Soil)	Nematode Infestation Parameters		Plant Fresh Weight	
	J2 and Eggs g^−1^ Roots	Gall Index (0–5)	Aerial Parts	Roots
	*C. camphora*			
50	23376 ± 379	4.2 ± 0.2	37.2 ± 0.2	9.4 ± 0.2
100	20579 ± 264	4.0 ± 0.0	40.2 ± 0.4	10.6 ± 0.2
200	12053 ± 179	2.8 ± 0.2	42.2 ± 0.4	11.0 ± 0.3
	*C. verum*			
50	12297 ± 259	2.4 ± 0.2	61.0 ± 0.5	10.4 ± 0.2
100	11945 ± 125	2.2 ± 0.2	70.6 ± 1.2	11.8 ± 0.2
200	9244 ± 198	2.0 ± 0.0	77.8 ± 2.3	14.2 ± 0.8
	*C. aurantium*			
50	21492 ± 244	5.0 ± 0.0	34.4 ± 0.2	8.8 ± 0.2
100	18685 ± 212	4.6 ± 0.2	37.4 ± 0.2	9.2 ± 0.2
200	16246 ± 244	3.8 ± 0.2	38.2 ± 0.5	9.4 ± 0.2
	*E. citriodora*			
50	15302 ± 168	4.0 ± 0.0	60.6 ± 1.4	13.0 ± 0.8
100	11222 ± 157	3.0 ± 0.0	72.8 ± 1.6	14.8 ± 0.7
200	9551 ± 209	2.2 ± 0.2	86.4 ± 3.1	18.4 ± 0.5
	*E. globulus*			
50	22321 ± 212	4.0 ± 0.0	40.2 ± 0.5	9.6 ± 0.2
100	16963 ± 219	3.2 ± 0.2	41.6 ± 0.5	10.6 ± 0.2
200	14938 ± 266	3.0 ± 0.0	42.2 ± 0.6	12.4 ± 0.2
	*M. piperita*			
50	20830 ± 484	3.8 ± 0.2	39.4 ± 0.4	9.8 ± 0.5
100	18137 ± 433	3.2 ± 0.2	40.4 ± 0.4	11.4 ± 0.2
200	14183 ± 335	2.4 ± 0.2	43.4 ± 0.2	12.6 ± 0.4
	*P. asperum*			
50	20197 ± 268	3.8 ± 0.2	60.2 ± 1.8	11.4 ± 0.2
100	15182 ± 307	2.6 ± 0.2	54.6 ± 0.9	10.8 ± 0.9
200	11738 ± 273	2.0 ± 0.0	47.4 ± 0.9	8.0 ± 0.3
	*R. graveolens*			
50	17871 ± 367	4.2 ± 0.4	53.6 ± 2.2	10.4 ± 0.7
100	15700 ± 458	2.8 ± 0.2	59.0 ± 2.9	11.6 ± 0.2
200	10126 ± 255	1.8 ± 0.2	67.4 ± 1.2	13.8 ± 0.2
	*S. molle*			
50	20368 ± 213	4.2 ± 0.4	43.8 ± 0.7	10.0 ± 0.3
100	16840 ± 203	3.0 ± 0.0	46.8 ± 0.8	11.0 ± 0.3
200	12419 ± 273	2.2 ± 0.2	49.8 ± 0.4	12.6 ± 0.2
	*S. aromaticum*			
50	12567 ± 131	3.0 ± 0.3	49.6 ± 2.2	9.6 ± 0.2
100	10633 ± 75	2.8 ± 0.4	61.4 ± 0.7	11.6 ± 0.6
200	9160 ± 94	2.2 ± 0.2	67.4 ± 1.7	13.4 ± 1.0
Oxamyl	7832 ± 275	2.0 ± 0.0	61.8 ± 0.9	11.6 ± 0.2
Tween20	28550 ± 1269	4.8 ± 0.2	40.8 ± 0.4	9.2 ± 0.2
Non treated	28318 ± 432	4.8 ± 0.2	41.0 ± 0.3	8.4 ± 0.4
Non infested	-	-	43.6 ± 0.2	10.4 ± 0.4
LSD (*p* = 0.05)	979	0.57	3.49	1.26
